# Clinician survey regarding measurable residual disease-guided decision-making in multiple myeloma

**DOI:** 10.1038/s41408-022-00705-6

**Published:** 2022-07-11

**Authors:** Benjamin A. Derman, Andrzej J. Jakubowiak, Michael A. Thompson

**Affiliations:** 1 Section of Hematology/Oncology, University of Chicago Medical Center, Chicago, IL USA; 2 Tempus Labs, Chicago, IL USA; 3 Aurora Cancer Care, Advocate Aurora Health, Milwaukee, WI USA

**Keywords:** Myeloma, Chemotherapy

Dear Editor,

Advances in multiple myeloma (MM) therapies have enhanced the likelihood of achieving deep and durable treatment responses which carry significant prognostic implications. Obtaining a complete response (CR) is an independent predictor of longer progression-free survival (PFS) and overall survival (OS) in MM [1–3]; however, more sensitive methods that assess for measurable residual disease (MRD) have been shown to further enhance prognostication in MM [4–7]. MRD status in MM is established as a prognostic biomarker during treatment, but its impact on real-world decision making remains unproven.

In a previous survey of clinician attitudes and practices toward MRD in MM, we found that in a respondent pool enriched for academic clinicians 91% reported assessing for MRD, 50% using an assay with a sensitivity threshold <10^−6^, and only 37% reported using MRD status to guide decision making [8]. This survey queried respondents for their practices in general but did not address specific scenarios to truly gauge *how* and *when* MRD might influence their management. Moreover, it was conducted in the months leading up to and just after the FDA approval of the clonoSEQ next generation sequencing assay for MRD [9], and it did not include sufficient representation outside academia.

In this study, we surveyed MM clinicians to understand their general attitudes toward MRD in MM and then if/how MRD status might impact their decision making using certain common clinical scenarios. We hypothesized that there would be a discrepancy between how respondents said they felt about using MRD to guide decision making in MM and how they responded to specific clinical scenarios.

An anonymous internet survey was distributed primarily through Twitter between November 2020 and December 2021 with the use of registered cancer ontology tags #mmsm and #mmMRD. The survey tool was approved by the institutional review board at the University of Chicago (supplement). In brief, the clinical scenarios for newly diagnosed MM included the following:

1. Post-induction therapy, standard-risk, transplant-eligible, MRD negative
2. Post-induction therapy, standard-risk, transplant-ineligible, MRD negative
3. Post-transplant, standard-risk, MRD positive
4. During maintenance therapy, standard-risk, sustained MRD negative
5. During maintenance therapy, standard-risk, MRD positive

For each scenario, respondents reported if and how their answer would be different based on changes in disease risk (i.e., high-risk) or MRD status.

There was a total of 90 respondents who agreed to participate; 89 (99%) completed the core survey, of which 68 (76%) submitted responses to the optional clinical scenarios section. Self-identified academic clinicians made up 76% of respondents (68/89) as opposed to a private practice or hybrid setting (21/89, 24%). Median clinical experience was 10 years as a hematologist/oncologist and 20 patients with MM per week (Table 1). Geographically, respondents were from North America (65%), Europe (23%), South America (8%), Asia (2%), and Australia (2%). Most clinicians (57/89, 64%) answered affirmatively to assessing for MRD in MM in a clinical (non-research) setting. There were no significant differences in demographics or background between MRD users and non-MRD users.

**Table 1:** Demographics and attitudes toward MRD in multiple myeloma.

Question	Clinicians not assessing for MRD in myeloma clinically (*n* = 32)	Clinicians assessing for MRD in myeloma clinically (*n* = 57)	All respondents (*n* = 89)
*Practice setting*			
Academic Health System	23 (72%)	45 (79%)	68 (76%)
Private Practice/Hybrid	9 (28%)	12 (21%)	21 (24%)
*Geographic location*			
North America	20 (63%)	38 (67%)	58 (65%)
Europe	3 (9%)	17 (30%)	20 (23%)
South America	5 (16%)	2 (4%)	7 (8%)
Australia	2 (6%)	0	2 (2%)
Asia	2 (6%)	0	2 (2%)
Africa	0	0	0
*Practice Experience, median (range)*			
Years in practice	10 (1–31)	10 (1–40)	10 (1–40)
Myeloma patient visits/week	15 (1–100)	20 (1–100)	20 (1–100)
Assess for MRD on Clinical Trials	12 (38%)	45 (79%)	57 (64%)
*Reasons for not clinically assessing for MRD*^a^			
Not able to order MRD test	7 (22%)	–	–
Unclear when to assess for MRD	7 (22%)	–	–
Not actionable result	6 (19%)	–	–
Cost/insurance coverage	5 (16%)	–	–
Discomfort of bone marrow aspiration	3 (9%)	–	–
Insufficient test sensitivity	1 (3%)	–	–
Not an appropriate surrogate endpoint	1 (3%)	–	–
Not familiar with MRD as an endpoint	0	–	–
*Concerns about MRD guiding decision making in myeloma*^a^			
No data to support decision making	14 (44%)	36 (63%)	50 (56%)
Unclear when to assess for MRD	12 (38%)	25 (44%)	37 (42%)
Discomfort of bone marrow aspiration	8 (25%)	14 (25%)	22 (25%)
Cost/insurance coverage	4 (13%)	18 (32%)	22 (25%)
Not a surrogate endpoint for OS	6 (19%)	12 (21%)	18 (20%)
Insufficient test sensitivity	3 (9%)	3 (5%)	6 (7%)
No concerns	3 (9%)	3 (5%)	6 (7%)

^a^Up to 3 answers allowed.

Of the 32 non-MRD users, the most common reasons (of 8 choices) for not using MRD were: inability to order MRD (22%), unclear when to assess for MRD (22%), lack of actionability (19%), and cost (16%) (Table 1). The most common (≥20%) areas of concern about using MRD status to guide decision making were (of 7 choices): no data to support decision making (44%), unknown appropriate timing (38%), and discomfort of bone marrow aspiration (25%). Of the 57 MRD users, the most common areas of concern were similar with a higher proportion (63%) stating there is no data to support decision making (Table 1).

We next assessed responses (*n* = 68) to the clinical scenarios previously discussed (Tables 2 and 3). For scenario #1 involving a transplant-eligible standard-risk patient with MRD negativity after induction, 15% (10/68) changed their answer to intensify therapy for high-risk disease and 16% (11/68) changed to intensify therapy for MRD positivity. Scenario #2 described a transplant-ineligible, standard-risk patient with MRD positivity after induction; 38% (26/68) changed their answer to intensify therapy if high-risk and 21% (14/68) would de-escalate therapy if MRD negative. When queried in scenario #3 about a patient with standard-risk disease after frontline autologous stem cell transplant (ASCT) who is MRD positive, 54% (37/68) offered a different response based on disease risk (intensify for high-risk disease) and 18% (12/68) changed their answer to de-escalate therapy if MRD negative. Scenario #4 assessed practices for a patient with sustained MRD negativity who has received maintenance lenalidomide for three years; 43% (29/68) answered that they would stop maintenance therapy; 28% (19/68) changed their answer to continue maintenance therapy in the setting of high-risk disease and 40% (27/68) changed their answer to continue therapy if MRD positive (scenario #5). Additionally, 16% (11/68) would switch therapy for patients with MRD positivity and high-risk disease in scenario #5.

**Table 2:** Responses to different clinical scenarios in first-line therapy for multiple myeloma.

MRD scenarios	Changed answer if high-risk cytogenetics (*n* = 68)	Changed answer if opposite MRD status (*n* = 68)
Post-induction, standard-risk, transplant-eligible, MRD negative	10 (15%)	11 (16%)
Post-induction, standard-risk, transplant-ineligible, MRD positive	26 (38%)	14 (21%)
Post-ASCT, standard-risk, MRD positive	37 (54%)	12 (18%)
During maintenance, standard-risk, sustained MRD negative	19 (28%)	27 (40%)
During maintenance, standard-risk, MRD positive	11 (16%)	N/A

*ASCT* autologous stem cell transplant, *MRD* measurable residual disease.

**Table 3:** Granular responses to different clinical scenarios in first-line therapy for multiple myeloma.

MRD scenarios	Responses (*n* = 68)	Action (intensify, de-escalate, continue)
*Post-induction, standard-risk, transplant-eligible, MRD negative*		
Proceed to ASCT	56 (82%)	**–**
Defer ASCT	10 (15%)	**–**
Would not evaluate	2 (3%)	**–**
Change answer if high-risk	10 (15%)	Intensify (ASCT)
Change answer if MRD positive	11 (16%)	Intensify (ASCT)
*Post-induction, standard-risk, transplant-ineligible, MRD positive*		
Continue same regimen (triplet)	19 (28%)	–
Change regimen	1 (1%)	–
De-escalate to single-agent maintenance	36 (53%)	–
Would not evaluate	12 (18%)	–
Change answer if high-risk	26 (38%)	Intensify (continue triplet)
Change answer if MRD negative	14 (21%)	De-escalate to single-agent maintenance
*Post-ASCT, standard-risk, MRD positive*		
Start triplet consolidation	11 (16%)	–
Start single-agent maintenance	52 (77%)	–
Tandem ASCT	2 (3%)	–
Would not evaluate	3 (4%)	–
Change answer if high-risk	37 (54%)	Intensify (triplet)
Change answer if MRD negative	12 (18%)	De-escalate to single-agent maintenance
*During maintenance, standard-risk, sustained MRD negative*		
Stop maintenance therapy	29 (43%)	–
Continue maintenance therapy	25 (37%)	–
Would not evaluate	13 (19%)	–
No response	1 (1%)	–
Change answer if high-risk	19 (28%)	Continue maintenance
*During maintenance, standard-risk, MRD positive*		
Switch therapy	2 (3%)	–
Continue maintenance therapy	46 (68%)	–
Would not evaluate	18 (26%)	–
Change answer if high-risk	11 (16%)	Intensify (switch therapy)
Change answer if sustained MRD negative	27 (40%)	Continue maintenance

*ASCT* autologous stem cell transplant, *MRD* measurable residual disease.

Analysis of responses to the clinical scenarios in aggregate reveal that 53/68 (78%) individual respondents would change at least one decision based on the presence of high-risk vs standard-risk disease, in favor of intensifying or continuing treatment in high-risk disease. A total of 41/68 (60%) individual respondents would change at least one decision based on an MRD result, and 37/68 (54%) used both disease risk and MRD status to make decisions. Importantly, of the 50 participants who responded that there is no data for MRD status to guide decision making, 27 (54%) ultimately answered a clinical scenario *differently* based on the MRD status. These findings suggest that while both disease risk and MRD status influence decision making in newly diagnosed MM, disease risk carries slightly greater significance (*p* = 0.04). That 60% of respondents reported using MRD status to guide decision making is surprising, considering that many of these respondents expressed a concern over the lack of data to use MRD status in this manner. This discrepancy points to an interesting phenomenon of the ‘insidious’/subconscious influence that MRD status has on decision making. It is also notable that the percentage of those using MRD to guide decision making rose from the 37% we reported in an earlier iteration of this survey [8] to 60% that we see in this current survey. We believe that this increase can be attributed to several phenomena: (1) The second survey used specific clinical scenarios to assess MRD use whereas the first did not, (2) increased dissemination of MRD-guided trial strategies, and (3) increased availability of a commercial centralized MRD assay in 2021 compared to 2018 (when the first was conducted).

Interestingly, MRD status led to decisions being made in a bidirectional fashion. MRD positivity led to clinicians seeking to intensify therapy, while MRD negativity led to de-escalation of therapy; this includes a strong preference for discontinuation of maintenance therapy in those with sustained MRD negativity and standard-risk disease.

Limitations of this study include a small sample size with the majority being academic clinicians, which may lean toward a higher propensity for usage of MRD testing in MM. The survey was primarily distributed by Twitter, making tracking of the survey response rate difficult to assess. Not all clinicians who treat myeloma are active on Twitter, so we cannot say whether our respondents were representative of the entire myeloma clinician community. This survey only covered scenarios involving newly diagnosed MM and is not exhaustive of every possible clinical decision point. The nuances in the clinical management of MM cannot be properly conveyed in such a survey.

The merits of whether MRD status should be used to guide decision making in MM remain hotly debated, especially considering that over half of respondents stated there is no data to support this strategy. The results of this survey suggest that MRD status is in fact subtly influencing decisions and serve as a catalyst for further investigation of MRD-guided treatment strategies in MM. There has thus far been only retrospective evidence that making clinical decisions based on MRD may improve outcomes [10]. Two trials have already used an MRD-adaptive approach to guide de-escalation of therapy with excellent short-term outcomes and long-term data still immature [11, 12]. Other ongoing studies, including MRD2STOP (NCT04108624) and SWOG S1803 (NCT04071457), will help answer whether maintenance therapy can be safely discontinued in patients with sustained MRD negativity. The EQUATE study (NCT04566328) is investigating the use of MRD to guide intensification of frontline therapy in those with MRD positivity in a randomized fashion. It will be vital to continue support and development of clinical trials with MRD-guided treatment schema to better inform its clinical utility in MM.

## Supplementary information

Complete Survey

## Data Availability

The datasets generated during and/or analyzed during the current study are available from the corresponding author on reasonable request.
