# Supplementary material for: Clinician survey regarding measurable residual disease-guided decision-making in multiple myeloma
Source: Blood Cancer J. 2022 Jul 11;12(7):108. doi: 10.1038/s41408-022-00705-6 (PMC9276661; doi:10.1038/s41408-022-00705-6)
Supplement: Supplementary file 1 — Complete Survey [file 41408_2022_705_MOESM1_ESM.pdf]

Survey: Use of MRD for Decision Making in Multiple Myeloma

You are being invited to participate in an anonymous survey titled "MODEMM: Use of Minimal Residual Disease for Decision Making in Multiple Myeloma". This survey is being conducted by Benjamin Derman, MD and Andrzej Jakubowiak, MD, PhD from the University of Chicago Medical Center and Michael Thompson, MD, PhD from Aurora Health Care. You were selected to participate in this survey because you are a clinician who treats patients with multiple myeloma.

The purpose of this research study is to better understand myeloma clinicians' practice habits and attitudes toward the use of minimal residual disease (MRD) for decision-making in multiple myeloma. If you agree to take part in this study, you will be asked to complete an online survey below. It will take you approximately 10 minutes to complete.

You may not directly benefit from this research; we hope that your participation in the study may help the myeloma community at large understand how MRD status is currently being used clinically in order to better inform research in this field.

We believe there are no known risks associated with this research study. To the best of our ability, your answers will remain confidential on a secure server. Your participation in this study is completely voluntary and you can withdraw at any time.

If you have questions or concerns about this project, contact bderman@medicine.bsd.uchicago.edu.

By clicking "I agree" below you are indicating that you are at least 18 years old, have read and understood this consent form and agree to participate in this research study.

	Do you agree to proceed?	◯ I agree<br>◯ I do not agree
1	Which of the following best describes the healthcare setting in which you work?	◯ Academic health system<br>◯ Private practice<br>◯ Hybrid model (private with academic affiliation)
2	In what area of the world do you practice medicine?	◯ Africa<br>◯ Asia<br>◯ Australia<br>◯ Europe<br>◯ North America<br>◯ South America
3	What is your degree?	◯ MD, DO, or equivalent<br>◯ MD,PhD<br>◯ PA<br>◯ NP (i.e. APN, DNP, or equivalent)<br>◯ Other
4	How many years have you been a practicing hematology/oncology clinician?	______________________<br>(Please provide a single number estimate)
5	Approximately how many multiple myeloma patients do you see per week?	______________________<br>(Please provide a single number estimate)

6	Do you use any of the following guidelines for decision making in multiple myeloma?	☐ NCCN<br>☐ US Oncology<br>☐ VIA Oncology/Elsevier ClinicalPath<br>☐ Atrium<br>☐ ESMO<br>☐ Other<br>☐ None of the above
	If 'Other', please list guidelines you use	__________________________
7	What is the extent of your participation in clinical trials specifically enrolling patients with multiple myeloma?	☐ Principal investigator (PI)<br>☐ Co-investigator<br>☐ None of the above<br>(Check all that apply)
8	Do you have patients enrolled on any clinical trials that use minimal residual disease as an endpoint?	○ Yes<br>○ No
9	Outside of clinical trials, are you assessing for minimal residual disease in any of your patients?	○ Yes<br>○ No

FOR RESPONDENTS WHO ANSWER ‘NO’ TO #9

10	You indicated that you are not assessing MRD status in your patients. What are your reason(s) for not assessing for MRD? Please select up to 3 choices.	☐ I am not familiar with the concept of MRD<br>☐ MRD is not an appropriate surrogate endpoint<br>☐ I don't know what to do with MRD status information<br>☐ I don't have the capability to measure MRD<br>☐ Cost/insurance coverage<br>☐ I don't want to subject my patient to another bone marrow biopsy<br>☐ The depth of my institution's MRD testing may be insufficient<br>☐ No consensus on the correct timing of MRD testing<br>☐ Other (please specify)<br>(Select up to 3 choices)

FOR RESPONDENTS WHO ANSWER ‘YES’ TO #9

10	What method(s) are you using to assess for minimal residual disease?	☐ Flow cytometry<br>☐ PCR (Polymerase chain reaction)<br>☐ NGS (Next generation sequencing)<br>☐ PET-CT<br>☐ MRI<br>☐ Other (please specify)<br>(Select all that apply)
	Please specify 'other':	__________________________
11	What is your PREFERRED method for assessing for MRD?	○ Flow cytometry<br>○ PCR (Polymerase chain reaction)<br>○ NGS (Next generation sequencing)<br>○ PET-CT<br>○ MRI<br>○ Other (please specify)
	Please specify 'other':	__________________________

12 What is the lower limit of detection (analytical sensitivity) of your preferred method of MRD testing?

- ◯ 10^-4 [1 aberrant plasma cell in 10,000 cells]
- ◯ 10^-5 [1 aberrant plasma cell in 100,00 cells]
- ◯ 10^-6 [1 aberrant plasma cell in 1,000,000 cells]
- ◯ I don't know

13 Do any of the following methods for MRD detection in myeloma have FDA approval?

- ☐ Flow Cytometry
- ☐ NGS [i.e. clonoSEQ, etc.]
- ☐ PET/CT
- ☐ MRI
- ☐ None

(Check all that apply)

FOR ALL RESPONDENTS

We will now present a series of 5 patient scenarios and we would like for you to respond with your thoughts on how you would manage these situations. (Estimated time per scenario: 1 minute)

14 A 56 year old woman (ECOG PS 0) was diagnosed 4 months ago with ISS 1, IgG Kappa Multiple Myeloma with standard-risk cytogenetics (trisomy 3, 5, and 7). She had measurable disease and a positive PET/CT at baseline. She has received 4 cycles of bortezomib, lenalidomide, and dexamethasone (VRd) and her SPEP/immunofixation shows no monoclonal protein and light chains have normalized. She presents for evaluation for potential high dose melphalan and autologous stem cell rescue. You decide to perform a bone marrow biopsy and aspiration with evaluation by your preferred method of MRD and PET/CT; both test results show MRD-negativity.

Question: After collection of stem cells, what do you advise the patient?

- ◯ Continue VRd and defer autologous transplant
- ◯ Proceed to autologous transplant
- ◯ Proceed to single agent maintenance therapy
- ◯ I would not have performed a bone marrow biopsy or PET/CT at this time

Would your answer differ if the patient had high risk disease?

- ◯ No
- ◯ Yes (explain)

Please explain briefly how your actions would be different.

____

Would your answer differ if the patient was MRD-positive?

- ◯ No
- ◯ Yes (explain)

Please explain briefly how your actions would be different.

____

15 A 78 year old man presented to you with ISS 2, standard risk cytogenetics, IgG Lambda Multiple Myeloma 6 months ago and started therapy with bortezomib, lenalidomide, and dexamethasone. He was deemed transplant ineligible and he just completed 8 cycles of treatment. He has not experienced significant toxicities from therapy. He currently has no detectable disease by serum markers. He undergoes a bone marrow biopsy with evaluation by your preferred MRD method and PET/CT, and the bone marrow MRD result returns positive for residual disease.

Question: What do you advise the patient?

- ◯ Continue VRd until toxicities emerge, then switch to maintenance at later point
- ◯ Switch to single-agent maintenance therapy
- ◯ Switch to alternative triplet therapy
- ◯ I would not perform a bone marrow biopsy or PET/CT in this scenario

---

Would your answer differ if the patient had high risk disease?

- ◯ No
- ◯ Yes (explain)

Please explain briefly how your actions would be different.

____

Would your answer differ if the patient was MRD-negative?

- ◯ No
- ◯ Yes (explain)

Please explain briefly how your actions would be different.

____

---

16 A 58 year old man (ECOG PS 1) with standard-risk ISS 1 (no high risk cytogenetics), lambda light chain only Multiple Myeloma received 5 cycles of induction therapy with bortezomib, lenalidomide, and dexamethasone (VRd). He then received high-dose melphalan and autologous stem cell rescue. At day +100 after his transplant, he has no detectable disease by serum or urine markers. He undergoes a bone marrow biopsy with your preferred MRD method, and the result returns positive for MRD.

Question: What do you advise the patient?

- ◯ Start VRd (or other triplet) consolidation
- ◯ Start single agent maintenance therapy
- ◯ Proceed with second (tandem) transplant
- ◯ I would not perform a bone marrow biopsy or PET/CT in this scenario

---

Would your answer differ if the patient had high risk disease?

- ◯ No
- ◯ Yes (explain)

Please explain briefly how your actions would be different.

____

Would your answer differ if the patient was MRD-negative?

- ◯ No
- ◯ Yes (explain)

Please explain briefly how your actions would be different. ____________________

17 A 62 year old female with ISS 2, IgG Kappa Multiple Myeloma with hyperdiploidy (standard risk) cytogenetics received 4 cycles of bortezomib, lenalidomide, and dexamethasone, followed by melphalan and autologous stem cell rescue, followed by maintenance lenalidomide. She has been on maintenance therapy for 3 years. She has grade 1 diarrhea from lenalidomide and intermittent cytopenias that previously required a dose reduction of lenalidomide. One year ago, a bone marrow biopsy with your preferred MRD evaluation and PET/CT were negative for disease. Her serum and urine myeloma markers are normal. She undergoes a repeat bone marrow biopsy with your preferred MRD evaluation and PET/CT and both results return negative for MRD.

Question: What do you advise the patient?

- ◯ Continue maintenance indefinitely
- ◯ Continue maintenance for a fixed duration if remains MRD-negative
- ◯ Stop maintenance therapy and start active surveillance
- ◯ I would not perform testing at this time (would not affect management)

Would your answer differ if the patient had high risk disease?

- ◯ No
- ◯ Yes (explain)

Please explain briefly how your actions would be different. ____________________

18 Consider a similar patient: A 62 year old female with ISS 2 IgG Kappa Multiple Myeloma with hyperdiploidy (standard risk) cytogenetics received 4 cycles of bortezomib, lenalidomide, and dexamethasone, followed by melphalan and autologous stem cell rescue, followed by maintenance lenalidomide. She has been on maintenance therapy for 3 years. One year ago, a bone marrow biopsy with your preferred MRD evaluation and PET/CT were negative for disease. Her serum and urine myeloma markers are normal. She undergoes a bone marrow biopsy with your preferred MRD evaluation and PET/CT and the bone marrow testing reveals MRD-positivity.

Question: What do you advise the patient?

- ◯ Continue current maintenance therapy
- ◯ Switch to alternative maintenance therapy
- ◯ Start multi-drug anti-myeloma therapy
- ◯ I would not perform testing at this time (would not affect management)

Would your answer differ if the patient had high risk disease?

- ◯ No
- ◯ Yes (explain)

Please explain briefly how your actions would be different. ____________________

19 Which of the following concerns do you still have about using MRD to guide your decision making in multiple myeloma? Please select up to 3 choices.

- ☐ I have no concerns
- ☐ MRD is not a surrogate endpoint for survival
- ☐ No data to support MRD in decision making
- ☐ Cost/insurance coverage
- ☐ Subjecting my patient to another bone marrow biopsy
- ☐ The depth of my institution's MRD testing is insufficient for me
- ☐ No consensus on the correct timing of MRD testing
- ☐ Other (please specify)

(Select up to 3 choices)

Please specify 'other':

____

Thank you for your time and your thoughtful responses. Please feel free to leave any comments, suggestions, or feedback.
